# Assessing the scalability of an integrated falls prevention service for community-dwelling older people: a mixed methods study

**DOI:** 10.1186/s12877-021-02717-6

**Published:** 2022-01-03

**Authors:** Susan Calnan, Karen Lee, Sheena McHugh

**Affiliations:** 1 School of Public Health, University College Cork, Western Road, Cork, Ireland; 2 School of Public Health, The University of Sydney, NSW Sydney, Australia

**Keywords:** Scalability, Scaling up, Falls prevention, Integrated care, Older people

## Abstract

**Background:**

There is growing acknowledgement of the need for a phased approach to scaling up health interventions, beginning with an assessment of ‘scalability’, that is, the capacity of an individual intervention to be scaled up. This study aims to assess the scalability of a multi-component integrated falls prevention service for community-dwelling older people and to examine the applicability of the Intervention Scalability Assessment Tool (ISAT). The ISAT consists of 10 domains for consideration when determining the scalability of an intervention, and each domain comprises a series of questions aimed at examining readiness for scale-up.

**Methods:**

Multiple methods were used sequentially as recommended by the ISAT: a review of policy documents, results from a service evaluation and falls-related literature; one-to-one interviews (*n* = 11) with key stakeholders involved in management and oversight of the service; and a follow-up online questionnaire (*n* = 10) with stakeholders to rate scalability and provide further feedback on reasons for their scores.

**Results:**

Three of the ISAT domains were rated highly by the participants. Analysis of the qualitative feedback and documents indicated that the issue of falls prevention among older people was of sufficient priority to warrant scale-up of the service and that the service aligned with national health policy priorities. Some participants also noted that benefits of the service could potentially outweigh costs through reduced hospital admissions and serious injuries such as hip fracture. The remaining domains received a moderate score from participants, however, indicating considerable barriers to scale-up. In the qualitative feedback, barriers identified included the perceived need for more healthcare staff to deliver components of the service, for additional infrastructure such as adequate room space, and for an integrated electronic patient management system linking primary and secondary care and to prevent duplication of services.

**Conclusions:**

Plans to scale up the service are currently under review given the practical barriers that need to be addressed. The ISAT provides a systematic and structured framework for examining the scalability of this multi-component falls prevention intervention, although the iterative nature of the process and detailed and technical nature of its questions require considerable time and knowledge of the service to complete.

**Supplementary Information:**

The online version contains supplementary material available at 10.1186/s12877-021-02717-6.

## Introduction

Scaling up effective interventions is considered important to maximise the impact on health outcomes and to respond to budgetary constraints in the health system [1–3]. The process of scaling up is defined as the “deliberate efforts to increase the impact of successfully tested health interventions so as to benefit more people and to foster policy and program development on a lasting basis” [4]. As a concept, scaling up is distinguished from routine adoption as it involves an explicit intent to expand the reach of an intervention to new settings or target groups and should be accompanied by a systematic strategy to achieve this objective [1, 5]. The scaling up of health interventions is a growing area in implementation research, with studies on the conditions that may hinder or facilitate implementation at scale emerging from diverse health issues and settings [1, 6–9].

Increasingly, the need for a phased approach to scaling up is acknowledged [10]. For scale-up to be successful, a number of steps are recommended: i) assessment of scalability of the intervention; ii) development of a scale-up strategy; iii) strategy implementation; iv) strategy evaluation; and v) assuring sustainability [11–13]. Regarding assessment of scalability, the first step in this process, scalability is defined as the “the ability of a health intervention shown to be efficacious on a small scale and/or under controlled conditions to be expanded under real world conditions to reach a greater proportion of the eligible population while retaining effectiveness” [14].

The emphasis on scalability as a precondition to scaling up has led to a growing interest in the development of tools to assess scalability. The Intervention Scalability Assessment Tool (ISAT) is a new scalability tool designed to assist researchers, practitioners, policymakers and programme managers to determine the scalability and/or readiness of a discrete health programme or intervention for scale-up [15, 16]. The assessment tool was developed through a review of implementation science literature and input from implementation researchers, policymakers and practitioners actively involved in programme management and/or the scaling up population health interventions and programmes [15, 16]. To date, there are few published examples applying scalability tools such as the ISAT in research and practice. A recent pilot study on the utility of the ISAT in making scalability decisions for real-world interventions found that the ISAT was perceived as a useful tool to assess the scalability of real-world health programmes, with only minor limitations and challenges cited by participants, such as the level of implied knowledge and skillset required to complete the assessment [17].

Given the limited evidence on assessing scalability, particularly in a high-income country context [17], and growing acknowledgment of the importance of assessing for scalability when planning for scale-up, this study aims to apply the ISAT framework to examine the scalability of a multi-component integrated falls prevention service for older people. Falls are one of the main causes of injury, physical incapacity and even death among older people [18]. Integrating care is proposed as a policy solution internationally to help address the significant health and social care needs of the ageing population [19], including older persons’ greater susceptibility to falls. The primary aim of the study is to ascertain the suitability of this integrated falls prevention service for scale-up from regional to national level on a phased basis. A second aim is to examine the feasibility and usefulness of applying the ISAT in the context of this real-world setting.

## Methods

### The intervention

The intervention of focus in this study is a multi-component integrated falls prevention service for community-dwelling older people implemented in southwest Ireland (Co. Cork) since 2015. The service provides a continuum of falls-related services across primary and secondary care, aimed at preventing, treating and managing falls and falls-related risks among the older population (aged 65+). The pathway service consists of a single point of access, managed by a dedicated falls coordinator, and it integrates existing and new services, including new falls risk assessment clinics delivered in primary care settings, which commenced in late 2015, approximately 5 years prior to the ISAT evaluation (see Supplementary file, S1 for TIDieR description of the service). Clinics were established in four primary care locations in the region (a further two clinics came on stream at a later point), and each clinic is delivered by a multidisciplinary team comprising a physiotherapist, occupational therapist and nurse. The service also consists of two consultant-led clinics providing specialist assessment and treatment in a hospital setting. The service is overseen by a steering group comprising health professionals and management working in older persons’ services and/or with an interest in falls prevention. Funding for the service was provided by Ireland’s national health service, the Health Service Executive (HSE).

### Study design

A mixed method study design was used to assess the scalability of this service, involving a combination of document analysis, qualitative interviews and an online survey. In line with Palinkas’ taxonomy of mixed methods approaches (based on a combination of previous taxonomies) [20], the ‘function’ in this context was ‘complementarity’ – that is, “using each set of methods to answer a related question or series of questions for purposes of evaluation”. The ‘structure’ entailed sequential collection and analysis of qualitative and quantitative data, beginning with qualitative data. Accordingly, document analysis and qualitative interviews were used to obtain background information for each domain of the scalability assessment. An online survey comprising pre-defined readiness assessment questions was then used to rate scalability. In the survey, participants were asked to choose categorical answers, with additional space provided for open-ended answers, an approach increasingly used in other studies [21]. The mixed methods ‘process’ focused on ‘connecting the data’, whereby one dataset seeks to build on another dataset.

Ethical approval to conduct the study was granted by the university social research ethics committee and all methods were performed in accordance with the relevant guidelines and regulations.

### Guiding framework

The ISAT provided the guiding framework, informing both data collection and analysis [15, 16]. The ISAT consists of 10 domains for consideration when determining the scalability of a service or intervention (see Supplementary file, S2 for overview of domains). Each domain comprises a series of questions designed to promote active consideration of factors deemed important when assessing scalability [15–17]. The 10 domains are organised into two parts: Part A (setting the scene) focuses on obtaining background information on the health problem of concern, on the context within which the intervention is proposed for scaleup, and on a description of the intervention; Part B (intervention implementation planning) focuses on implementation and feasibility factors relating to all aspects, including fidelity and adaptations, reach and acceptability, delivery settings and agents, as well as implementation infrastructure and training. A final Part C creates a summative assessment based on a series of readiness assessment questions posed following completion of Parts A and B. The results of this summative assessment are used to generate a radar plot against which readiness for scale-up can be assessed, and to prompt a final recommendation regarding the suitability of the intervention for scale-up.

### Data collection and analysis

**Fig. 1:**
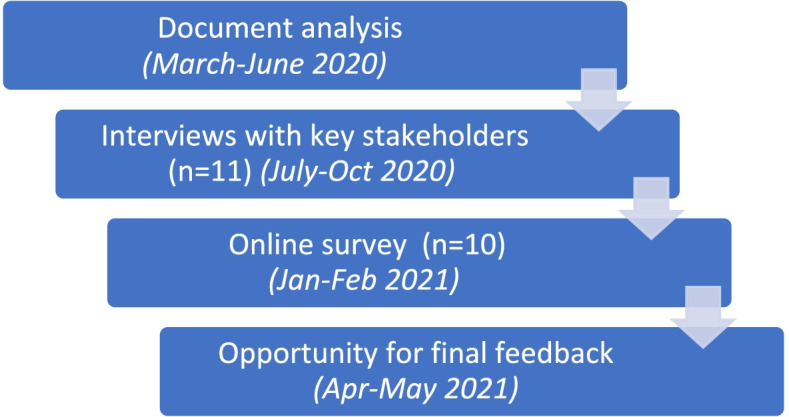
Main steps in data collection and analysis for study

Data collection and analysis involved four main steps devised by the research team based on the ISAT guidance, which recommends using a variety of sources to complete the assessment [15, 16]. A researcher (KL) who was involved in developing the ISAT also provided guidance throughout the study. Four steps were undertaken iteratively to complete the ISAT: 1) a document analysis of policy documents, service data and falls-related literature; 2) interviews with key stakeholders involved in the management and establishment of the service; 3) a follow-up online questionnaire; and 4) presentation of results with final opportunity for feedback (Fig. 1). Data analysis for this study involved a combination of document analysis, qualitative content analysis and quantitative analysis. An overview of the data collection and analysis for each step is provided below.

#### Step 1: review of existing data and literature

A document analysis was undertaken to answer specific questions raised in the ISAT. Document analysis is a form of indirect data analysis useful for exploring texts such as policy documents, legislation and protocols, as distinct from a literature review, which is largely a review of research studies conducted by past researchers [22]. The document analysis conformed to O’Leary’s ([22], p. 499) eight-step approach, which includes gathering the relevant texts, organising, confirming their authenticity and finally exploring the content.

Researchers (SC, SMH) compiled a list of appropriate data sources for the corresponding questions in the ISAT. Four types of document were included in the review, as detailed in Table 1 below.

**Table 1:** Types of documents used in the analysis

Type of document	Details
Service documents (unpublished)	- Project initiation document - Evaluation report on service (including results of interviews conducted with service providers delivering clinics)
Policy documents	- International [23], national [24, 25] and regional policy documents [26] on falls prevention in older people - National health policy literature [27–29]
Clinical guidance	- National Institute for Health and Care Excellence (NICE) guidelines [30, 31] - American Geriatrics Society/British Geriatrics Society guidelines [32] - Health Quality and Safety Commission in New Zealand review (2020) [33]
General falls literature	- Research on prevalence [23, 34–38] and impact of falls [34, 39–47] on older people, and on effectiveness of falls interventions [48–54]

For the initial exploration phase, an ‘interview technique’ approach was used, whereby the researcher ‘asked questions’ of the text (in the sense that you are treating each document as a respondent who can provide information relevant to your enquiry), highlighting relevant passages of the text to answer the question ([22], p. 498). For this study, the questions posed were those outlined in the ISAT – particularly contextual type questions regarding the health issue of concern, the wider strategic/political context, details of the intervention and evidence of effectiveness. Relevant information from these documents was highlighted to populate the corresponding sections of the ISAT and then summarised across the sources.

#### Step 2: stakeholder interviews

One-to-one interviews were conducted with a purposeful sample of key stakeholders involved in the implementation and oversight of this service. The objective of the interviews was to provide further information to answer questions posed in the ISAT, including details of the intervention and information for Part B (intervention implementation planning). To identify and select information rich cases with knowledge of implementation, criterion sampling was used, whereby participants were selected according to a predetermined criterion of importance [55].

In this instance, the criterion of importance was involvement in setting up, managing and/or overseeing the integrated falls prevention service for the region in question, and all interviewees were in senior positions and members of the service’s steering group. The reason for selecting senior stakeholders (as opposed to healthcare professionals directly involved in service provision) was that the ISAT evaluation required detailed knowledge of the underlying impetus for the service and details of its establishment and oversight, information which senior stakeholders were best placed to provide, in line with the criterion of identifying ‘information rich cases’. In addition, the stakeholders taking part were those who would be advising on decisions about scale-up. It should be noted also that the views of healthcare providers were captured in a separate study to this on perceived barriers and facilitators to implementation; while that study did not address scalability specifically, there were similarities between both studies in some of the issues raised, particularly regarding resource and infrastructure needs.

The interviewees for this study comprised health service management, geriatric consultants, service coordinators and heads of discipline in primary/community care (occupational therapy, physiotherapy, nursing). A total of 12 interviewees were invited to take part via an email invite, which was followed up by a phone call to confirm participation.

Interviews were carried out (by SC) over a three-and-a-half-month period (mid-July to late October 2020). The topic guide (see Supplementary file, S3 for interview questions) was created based on questions outlined in the ISAT, particularly those that could not be readily answered by the document analysis. The interviews were conducted mainly by phone (one was conducted online) based on the availability and preferences of interviewees – largely influenced by the restrictions arising from the Covid-19 global pandemic during the research period. The interviews were recorded using a digital recording device and transcribed professionally.

For analysis of the one-to-one interviews, qualitative content analysis was used, a “systematic classification process of coding and identifying themes or patterns” ([56], p. 1278). In this study, transcribed interviews were coded deductively in NVivo according to category nodes created to correspond to the ISAT questions in the interviews. A comprehensive summary of the findings from the document analysis and qualitative content analysis was then compiled to inform step 3 (online questionnaire) of the process. A second researcher (SMH) reviewed the preliminary results. Queries raised were then discussed by both researchers (SC, SMH) and the summary was updated accordingly.

#### Step 3: online questionnaire to rate scalability

In the third step, an online follow-up questionnaire was disseminated to participants to rate scalability of the service (January–February 2021). The questionnaire was circulated to the same participants who took part in step 2. ISAT guidance recommends conducting this part of the assessment in person as a group; however, this was not possible at the time due to the aforementioned restrictions of Covid-19. As an alternative, additional open-ended feedback sections were included in the survey for participants to give more detailed feedback alongside the single-choice questions.

The questionnaire was based on the readiness assessment questions provided in the ISAT – a series of single-choice questions (19 in total, see Supplementary file, S4) designed to assess for readiness to scale-up [16]. For each domain, a condensed summary of the results from the document analysis and interviews was provided and participants were asked to rate scalability given the evidence for that domain. The questions were rated using a four-point scale (not at all (0), to a very small extent (1), somewhat (2), to a large extent (3)) and corresponded to specific sections of the ISAT. Participants were invited to provide feedback on why they selected the particular option for each question/set of questions. Two additional questions were added regarding the scalability of the service in the context of Covid-19.

Prior to its dissemination to participants, the survey was piloted by a healthcare professional not attached to the study. For analysis of the survey, responses were scored according to the system outlined in the ISAT guidance [16]. Accordingly, each question was scored from 0 to 3 using the four-point scale outlined above. As there were multiple participants, the total score per question was totalled and averaged across the number of scorers, as advised by the ISAT guidance (e.g. a total score of 22 with 10 participants gives an average score of 2.2). In order to derive a final score for the domain, the average score across the questions (if there is more than one question in that domain) was calculated. The minimum score per domain was 0 and maximum score was 3. The final scores for each domain were subsequently inserted into the prescribed ISAT scoresheet (in Excel) [57]. This scoresheet created a visual representation (radar plot) of the final scores, thus illustrating the summation of the participants’ views on each domain. The resulting plot highlighted the domains that need to be strengthened or improved. In addition to this radar plot, data in the feedback sections of the survey were reviewed for each domain of the ISAT.

#### Step 4: presentation of results and opportunity for feedback

In a final step, a ‘scalability report’ containing the radar plot (visual representation of the final scores), a synopsis of information gathered for each domain of the ISAT and a conclusion on overall scalability of the service was compiled. Study participants were invited to an online meeting at which mains results of the study (including radar plot) were presented (in PowerPoint format). A two-page research brief of the findings was also disseminated to the participants after the meeting*.* This gave participants the opportunity to provide final feedback and comments on the results. As only minor comments were received from the participants, the results outlined here are largely similar to those outlined in the presentation and research brief.

## Results

### Participants

A total of 11 out of 12 stakeholders invited (92%) took part in the one-to-one interviews. Interview participants comprised senior stakeholders involved in management and oversight of the service, all of whom were members of the steering committee, as follows: health service management (2), geriatric consultants (2), service coordinators (2) and heads of discipline in primary/community care (5). Average length of interviews was approximately 45 min. In the second part of the study (the online survey), the same participants were invited to participate: 10 of the 11 (91%) original interviewees took part.

### Assessment of scalability

**Fig. 2:**
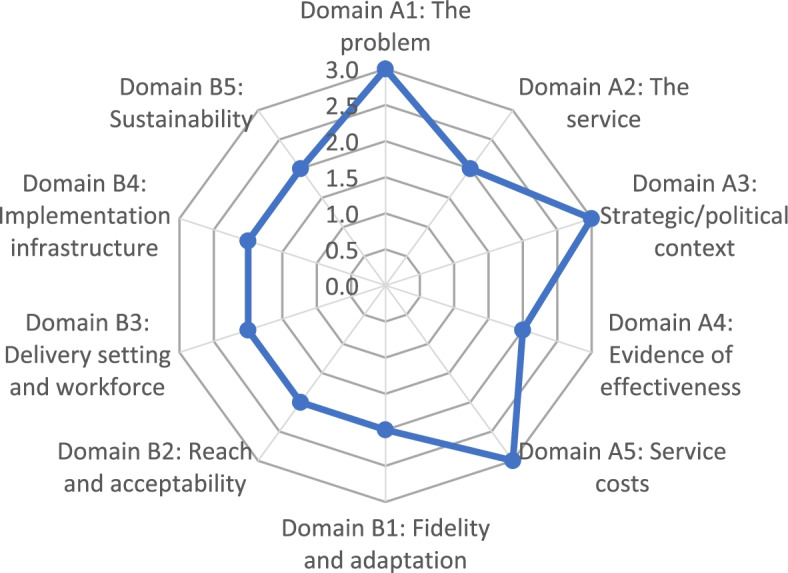
Results of the ISAT radar plot

The radar plot in Fig. 2 shows the results of the scalability assessment based on the responses received in the online survey. Points on the outer part of the radar plot below indicated domains that received a higher score and that were deemed more favourably; points closer to the centre indicated weaker areas or areas requiring further consideration. The results of the radar plot showed that the problem being addressed (risk and prevalence of falls among older people), the strategic/policy context and service cost received a high score. The remaining domains received a moderate score, indicating areas requiring greater consideration or further information, as elaborated in the sections below. Many of these domains, albeit not all, related to Part B of the ISAT, focused on implementation and feasibility factors.

### ISAT domains

#### Domain A1: whether the problem of falls is of sufficient priority to warrant scale-up

This domain scored highly (3.0) in the online survey, with all participants agreeing that the issue of falls prevention and management among older people was of sufficient priority to warrant scale-up of this service. Results of the documentary analysis supported this finding, underlining the higher prevalence of falls among older people and increased need for falls prevention and management services given the rising ageing population. In the qualitative feedback (interviews and online), the favourable response for this domain was attributed to a range of reasons: the potentially detrimental consequences of falls for older people; the high proportion of falls referrals experienced; and the need for and benefits of a more coordinated and appropriate service to prevent and treat falls, and identify risks, among this cohort. Other reasons cited included the growing ageing population; the need for a community response to help reduce more complex presentations, such as hip fractures; and the underreporting of falls.

#### Domain A2: whether intended outcomes of the service will address the needs of target population

While there was strong recognition of the need for a coordinated falls service in this study, only a moderate score (2.0) was achieved overall when participants were asked whether they believed the outcomes intended by this service would address the needs of the target group (65+ years) and problem of falls and falls-related harms. Reasons cited for this response in the qualitative feedback (interviews and online) included the need for additional educational, assessment and treatment elements to be added to the pathway (e.g. bone health information, fracture liaison services); the need to increase capacity in primary care in terms of delivery staff and service provision (e.g. more falls risk assessment clinics) and to further develop community falls-related services (e.g. strength and balance exercise classes, signposting to other services), given their potential to target a wider cohort (including the well older person). The complex needs of many people at risk of falls were also underlined in the feedback, as was the need for further research on the effectiveness of complex falls interventions. The challenges related to Covid-19, which prevented full operation of the service (e.g. temporary suspension of falls assessment clinics), were also cited as a barrier to delivering intended outcomes.

#### Domain A3: alignment of service with the wider strategic/political context

The service’s alignment with the strategic/political context received a high score (3.0) in the ISAT assessment, and participants believed that the service would be strategically useful to its funders (i.e. HSE). In the survey feedback, participants commented that providing an appropriate and coordinated service for older people is fully consistent with the policy and strategic direction of the health service and health nationally (“*falls is one of the geriatric giants*”). This finding supported those of the documentary analysis, which noted an increasing emphasis on falls prevention and integrated care in national health strategy documents: for example, falls prevention and management is listed as one of the 10 areas for development under the health service’s Integrated Care Programme for Older Persons in Ireland [29].

In both the interviews and survey feedback, participants commented on the potential transferability of and learning from this model of care, which integrated existing and new services, as well as formally linking primary and secondary care falls services. One participant noted that falls prevention services were well developed in the region and could provide valuable learning to other parts of the country, where services were perceived as being less well developed. Other possible reasons for the favourable assessment of this domain included the perceived cost savings that could be generated from a more integrated falls prevention service through reduced hospital/ED admissions; and the growing recognition of the need to address falls among older people owing to the rising ageing population, as highlighted in both the interviews and survey feedback.

#### Domain A4: effectiveness of service in addressing the problem of falls

On the question of whether the service would be effective in addressing the problem of falls in the target population (older people aged 65+), this domain received a moderate score (2.0) overall in the ISAT assessment. A range of reasons were put forward for this response in the qualitative feedback, including: the perceived need for a greater focus on falls prevention (especially exercise initiatives) within the pathway rather than solely assessing/treating those who have already fallen; the need for further research to evaluate the service (i.e. research to evaluate the effectiveness of this specific service); and the mixed results on the effectiveness of multifactorial falls interventions in existing research. The documentary analysis also underlined the mixed results regarding falls prevention interventions: for example, while the evidence regarding exercise interventions remains strong, the evidence on multifactorial interventions is more variable and suggests they may not be as effective as previously thought.

#### Domain A5: service costs and benefits

Regarding the question of whether benefits of the service could potentially outweigh the costs of the service, this domain received a high score (3.0) overall in the ISAT assessment. Results of the qualitative feedback (interviews and online) indicated that the cost of the pathway was perceived to be relatively low, with potential high cost savings from reducing the number of hospital admissions and risk of hip fractures, for example. The perceived large number of assessments undertaken by the service and relatively low level of investment were cited as indicators that benefits could outweigh costs. The psychosocial support provided to older persons attending the service was also perceived to be an important and long-term benefit. However, participants underlined the need for research to assess if the benefits of the service outweigh the costs. Budgetary constraints in terms of hiring new staff were cited as an ongoing pressure facing the country’s health services in the qualitative feedback for this study.

#### Domain B1: fidelity and adaptation of service at scale

In relation to maintaining/monitoring fidelity and the impact of adaptations to the service at scale, this domain received a moderate score (2.0) in the online survey. While there was greater consensus on two of the three questions in this domain, the perceived impact of adaptations to the core components of the service during scale-up generated a more variable score.

The variation in how each primary care service is delivered was cited as a challenge in the qualitative feedback, making it difficult to replicate the service in other primary care areas. In the interviews, it was noted that the real value of the service was the blueprint or ‘design principles’ it would provide to other regions on how to develop and implement a service of this nature, suggesting that it is the ‘function’ rather than ‘form’ that should be replicated. The need for an integrated IT system linking primary and secondary care services was also highlighted in the interviews and survey feedback to enable the monitoring of progress and avoid duplication of services in the pathway. Having a dedicated falls coordinator and steering group were also underlined as important attributes of the current service that should be maintained at scale-up to ensure monitoring and oversight.

#### Domain B2: reach and acceptability of the service to the target population

Regarding service reach and acceptability, this domain also scored moderately (2.0) in the ISAT assessment. The qualitative feedback provided further insight into possible reasons for this score. In terms of service reach, participants emphasised in the interviews the need to prevent, manage and treat falls among those at a lower level of complexity rather than solely for those presenting at the specialist level to ensure that older persons *at risk* of falling are identified and supported early on. The potential to widen the reach of the service to also target people aged 50–65 for falls prevention (e.g. strength and balance exercise classes) and bone health protection was also raised. Greater resourcing of the primary care services on the falls pathway was once again highlighted in this context to enable wider service reach. The need to increase awareness of falls prevention and services among the older population was also noted in the interviews through such measures as a national website and public education campaign.

In terms of acceptability of the service to the target population, service users’ desire for greater access to follow-up interventions following assessment was noted in the survey feedback (reflecting findings of the service evaluation). The need to monitor service user acceptability and uptake at every stage along the pathway was also noted. Factors perceived to positively influence acceptability included service users’ appreciation and experience of the benefits of multidisciplinary working.

#### Domain B3: delivery workforce and setting

A moderate score (2.0) was received for the domain on delivery workforce and delivery setting. The benefits of multidisciplinary working were noted in the interviews, and in the online feedback it was described as the future model of care in healthcare services which was likely to become an increasingly acceptable way of working for healthcare professionals. However, in both the interviews and online feedback, participants emphasised the challenges related to adequate staffing and coordination of the falls risk assessment clinics in particular. The lack of resources and increased time pressure on healthcare professionals delivering these clinics were cited as potential factors reducing the acceptability of the clinics to service providers, and this was supported by the documentary evidence which noted healthcare professionals’ dissatisfaction with inadequate resourcing of the service in an earlier evaluation. The need for greater ownership of and support for the service by primary care leadership, including prioritisation of resources, was highlighted for the service to be scalable. While the role of primary care services in prevention and assessment of falls was deemed crucial, the issue of managing complex falls cases in preventative assessment clinics was cited as a possible source of frustration for primary care staff.

#### Domain B4: implementation infrastructure

In terms of the feasibility of the implementation infrastructure required for scale-up, this domain scored moderately (2.0) in the ISAT assessment. In both the interviews and online feedback, lack of room space for assessment clinics was cited as an issue at some sites. The need for an integrated electronic patient management system for the service, linking primary, secondary and acute services, was once again highlighted and described as a key challenge facing the existing service, leading to a lack of communication between parts of the service and the potential for greater duplication of services. In the online feedback, the challenge of securing the appropriate infrastructure that meets Covid-19 standards was further underlined. The need/importance of strategic leadership and commitment in acquiring the required infrastructure was also highlighted, and it was argued that the necessary infrastructure could be organised if the will was there among health service management. The need for additional infrastructure for older persons’ services was underlined given the rising ageing population.

#### Domain B5: sustainability

The final domain of the ISAT assessment addressed the perceived sustainability of the service in terms of the level of resourcing and/or integration into delivery settings required for implementation at scale, as well as the potential sustainability of the delivery workforce (both service delivery and coordination staff). Similar to the previous domains, this domain received a moderate score (2.0) overall in the ISAT assessment.

In the survey feedback, participants further emphasised the need for increased and dedicated staffing in primary and secondary care to sustain the service in the long term, as was similarly raised in the interviews. The redeployment of healthcare professionals during Covid-19 and postponement of the assessment clinics was cited as challenge in both the interviews and online feedback for current service sustainability, although there was optimism that this would diminish with the roll-out of vaccination programmes. Plans nationally for significant investment in community services and multidisciplinary teams was highlighted as having the potential to positively impact on the scalability and sustainability of this service in the future. The reconfiguration of primary healthcare services to a community healthcare network model was also cited as a significant development, and it was argued that falls prevention services should be a cornerstone of older persons’ services at a network level. The community healthcare network model aims to respond to the significant challenges facing the Irish healthcare system, including the rising ageing population, by strengthening primary care teamworking and localising decision making so that decisions can be made closer to the point of care and specific to population needs within the network [58]. The increased emphasis on primary care services and prevention was further supported by the documentary evidence for this study, most notably the country’s 10-year policy roadmap for health reform, which highlights the importance of “patients accessing care at the most appropriate, cost-effective level with a strong emphasis on prevention and public health” [27].

## Discussion

The aim of this study was to assess the scalability of a multi-component integrated falls prevention pathway. The assessment suggests that while some aspects of scalability support the potential scale-up of the service based on the high scores received, the moderate scores received in other domains, particularly those pertaining to implementation and feasibility, raised questions over the suitability of the service for scaling up in its current form.

Overall, the problem of falls among older people was deemed of sufficient priority to warrant scale-up of this service, and the service was perceived as having the potential to provide a blueprint or ‘design principles’ for other regions planning to implement a falls prevention pathway. The perceived potential for cost-savings arising from the service was also highlighted – for example, due to reduced hospital admissions and hip fractures owing to improved falls prevention services – although robust evidence of cost-effectiveness would be required to substantiate this perspective. These drivers of scalability suggest that the service is of potential strategic importance for informing the development of falls prevention services, providing valuable learning for future services of this kind and in line with the growing emphasis on developing integrated care pathways and primary care services at national health policy level [27]. However, the lower score received in other domains highlights the importance of viewing scalability from a holistic perspective: while the service may align with the health policy context and address the important issue of falls prevention, scalability may be undermined by key evidence and resource gaps that need to be addressed before deciding to scale up.

The gap in evidence on the effectiveness of this specific service was highlighted as a limitation by stakeholders in this study, as were the mixed results regarding the effectiveness of falls interventions in existing research. Evidence supporting exercise for falls prevention remains strong [52], for instance, whereas evidence on multifactorial interventions is more varied [31, 49]. While the relative newness of this falls prevention pathway is a possible driver of stakeholders’ desire for effectiveness data, the lack of focus on effectiveness in scale-up research more broadly is borne out in other studies: for example, effectiveness is cited as one of the key areas receiving insufficient attention in the scaling up of health promotion interventions [14]. A recent review of studies on the scaling up of chronic disease prevention interventions showed that 15% of the scaled-up programmes were based on no discernible evidence of efficacy/effectiveness [59]. Moreover, decisions to scale up are not driven by evidence or research processes alone: factors such as political need, strategic context, funding availability and influence of key actors also play a role [60]. Scalability researchers strongly recommend that only efficacious/effective interventions should be scaled, highlighting the risk of scaling up interventions that do not work and diverting scarce resources away from potentially effective interventions [15]. Given the importance of effectiveness data in assessing for scalability, determining the effectiveness of this particular service, and robust evidence to support individual components of the pathway particularly new elements, will be crucial before deciding to scale up. Using a hybrid effectiveness-implementation study design [61] would be worth exploring for future research on services of this kind that may be considering scale-up.

Greater consideration of the effectiveness of the service cannot be viewed in isolation from key resource gaps, since if the service is not implemented properly with adequate resourcing, it is likely to have an impact on its long-term effectiveness in terms of service delivery and patient outcomes. The importance placed on primary care and prevention in this study reflects both international and national trends. In Ireland, the goal of developing primary care centres and teams has been a cornerstone of healthcare policy in recent decades [62], with an emphasis on community health networks (comprising 4–6 primary care teams) and ambulatory care hubs emerging in more recent years, as underlined in the country’s 10-year policy roadmap for healthcare reform, Sláintecare [27]. However, the study also underlines the need for better resourcing of primary care in terms of delivery workforce and implementation infrastructure for services such as this falls prevention pathway to be scalable and to help ensure service reach. Such findings reflect broader research on Ireland’s healthcare system, which points to a disjoint between the policy emphasis on primary care as the central focus of healthcare delivery and concrete plans and resources for its implementation processes, hindering progress on Ireland’s ‘top down’ primary care policy [62].

Regarding delivery workforce, the perceived need for more and dedicated staff to deliver components of the integrated falls service was repeatedly highlighted by participants in this study. Moreover, there was a sense that healthcare professionals perceived the service as an addition to their existing workload, which is not surprising given the service largely relied on existing workforce capacity to deliver the service. Challenges related to the implementation infrastructure were also raised, including the lack of adequate room space for assessment clinics at some implementation sites. The importance of strategic leadership and commitment in acquiring the required infrastructure at health service management level was also underlined. These findings echo those of Norton and Mittman, who found that key barriers to scaling up health prevention programmes included reluctance by implementing organisations to fully integrate programmes into routine service delivery on top of existing workloads and a lack of resources to implement programmes with fidelity or at all in ‘real-world’ settings [63]. The results of this study also reflect the reality of budgetary constraints within healthcare systems, including austerity cuts following periods of economic recession, which have resulted in a significant gap between expected and actual funding for human resources and infrastructure for the health service, including primary care [62]. One idea raised in this study to address delivery workforce constraints was the possibility of substituting certain roles in the service if scaled up to other regions: for instance, allocating senior nursing practitioner roles in place of geriatric consultants in regions where the latter were not available. Many falls prevention exercise interventions tested in RCTs were originally designed to be delivered by allied health professionals and have subsequently been delivered successfully by alternate workforces that meet competency standards at substantially reduced costs [14]. This approach aligns with Milat et al’s [14] emphasis on the need for “lateral responses” when scaling up interventions to respond to human resource capacity constraints.

While the emphasis on adaptation in scalability research to ensure good fit with local needs and circumstances [59, 64] is borne out in this study, balancing the competing pressures of fidelity and adaptation is acknowledged as an inherent challenge in scaling up or replicating interventions [14, 65]. In this study, although the need for flexibility to adapt was acknowledged, challenges related to monitoring fidelity were also highlighted. In particular, the lack of an integrated electronic patient management system linking disparate elements of the health service (e.g. primary and secondary care, emergency departments) was cited as key barrier, leading to fragmentation, potential duplication of referrals and lack of communication between different parts of the service. This in turn impacted on perceived fidelity as service providers were unable to readily track inward and outward referrals from other parts of the service, including ascertaining whether patients received the follow-up treatment or intervention prescribed in the falls assessment. The lack of interoperable electronic health records (EHRs) for patients is cited as a common shortcoming facing healthcare systems in many countries including Ireland. Shull [66] highlights that while EHRs are “the connective tissue of a health system”, most countries have systems that cannot unite the information of all their citizens because the software used in one part of the healthcare system may be incompatible with that used in another part. Fennelly et al. [67] in their umbrella review underline the value of EHRs, which provide a longitudinal record of information on an individual’s health status in computer-processible form across practices and specialists and enable authorised access to clinical records in real-time. EHRs not only increase the capacity to use clinical data for monitoring patient outcomes and conducting audits and research, the authors outline, but also provide access to patient information in a timely manner, enabling healthcare professionals to spend more time with patients, reducing duplication of tests and work, and improving the safety and quality of care provided. However, key organisational, human and technological factors also need to be addressed for the successful implementation of interoperable EHRs [67], underlining the importance of adequate planning and research in applying such systems. The need for and value placed on creating an integrated patient management system in this study highlights that technical and not just workforce requirements are among the key resources needed to enable scale-up of integrated care interventions [14, 64]. Technological infrastructure, along with other infrastructural and human resources requirements, should be subject to robust planning to ensure future sustainability of such interventions.

### Usefulness and feasibility of the ISAT

In this study, the ISAT provided a systematic and transparent approach for assessing scalability, enabling a thorough assessment of the diverse range of issues for consideration prior to scale-up.

While the ISAT is not designed as a psychometric tool to assess the interactions between the domains [15], a valuable lesson learnt from application of the ISAT is the importance of viewing scalability from a holistic perspective. From this study, it was apparent that no single factor alone should determine scalability, and any decision to scale up should first give due consideration to the complex interplay of factors detailed in the ISAT framework. This was supported by the developers of the ISAT, who indicate that the tool should be applied pragmatically and flexibly to allow for users to make their own considerations within their own contexts, including the priorities placed on one domain over the other when considering the decision to scale-up or not [15, 17].

Inclusion of the radar plot was particularly beneficial for communicating with stakeholders, providing a visual representation of areas that need to be strengthened or that require further discussion. These findings reflect preliminary research on application of the ISAT, which indicates that it was perceived as a useful tool for assessing the scalability of real-world health programmes and that it provided a structured process for making a decision on scalability [17].

One challenge experienced was the time and number of steps required to complete the ISAT, which involved a considerable amount of data collection and analysis (document analysis, interviews and online survey). The lack of information in some areas (e.g. effectiveness data) also proved challenging, although in this study it served to highlight aspects of the service requiring greater consideration before deciding to scale up. Other studies have raised questions over whether the ISAT may be less applicable for interventions with an emerging or unclear evidence base, and that it largely assumes a research-driven process of scaling up [15, 17]. Moreover, those with less experience with the concepts and requirements of scalability could potentially find the ISAT challenging to complete [15]. In this study, the stakeholders taking part in the study were all senior health professionals/managers and therefore well placed to answer questions related to scalability with the researcher’s support. The study also benefitted from the input of a researcher who was one of the lead authors in the development and testing of the ISAT. For managers or healthcare professionals using this tool without researcher input, however, there may be a need for additional support including greater explanation of technical terms and synthesis of information from multiple sources. We did not undertake a formal assessment of its perceived appropriateness and feasibility among practitioners; thus, further research on its usability in both research and non-research settings would be worthwhile.

### Limitations

Limitations of the research include the fact that study participants comprised stakeholders tasked with managing/overseeing the service but not healthcare practitioners involved in delivering the service. Therefore, healthcare practitioner perspectives on scalability were not included and may have led to different conclusions. A further limitation is the breadth of stakeholders involved in this study compared with all of those involved in delivery, and in this context the scores and feedback should be viewed as a guide only.

Challenges arising from the Covid-19 pandemic precluded the opportunity to conduct an in-person focus group with study participants, which is recommended as one of the steps in completing the ISAT. However, use of an online questionnaire with the option to give open-ended feedback and providing an online presentation of the main findings to participants, as well as disseminating a two-page research brief, ensured that they had sufficient opportunity to give feedback and that they were presented with evidence to support and/or challenge beliefs. A further limitation of the online survey feedback versus in-person focus group is that participants do not have the chance to engage in robust discussions with each other in real-time, which may have yielded additional information or even resulted in different scores. An opportunity for follow-up group engagement (e.g. via an online meeting or by circulating a draft report to the group for feedback) may have been valuable in the future to facilitate further debate and discussion around the results presented.

## Conclusions

While scale-up of this fall prevention service may be merited due to the prevalence of falls among older people and alignment of the service with the health policy context, current scalability is under review given the barriers that need to be addressed. Improved resourcing, particularly in primary care, and establishing an integrated electronic patient management system are among the recommendations to enable future scale-up of this service to other regions in the country. The gap in evidence of effectiveness of the service is a further limitation that needs to be addressed before scale-up can be recommended. The ISAT provides a pragmatic, yet systematic and structured framework for examining scalability in this context, although the detailed and technical nature of its questions require considerable time and knowledge of the service in order to complete.

## Supplementary Information

**Additional file 1: S1.** TIDieR-PHP intervention description. **S2.** Intervention Scalability Tool (ISAT) domains and objectives. **S3.** Interview questions. **S4.** Readiness assessment questions in online survey.

## Data Availability

The dataset for this study will not be shared given the small number of participants and specific nature of the service; also interviews were only part of this study. However, we have provided a supplementary file including details such as questions used in the interviews and online questionnaire and a detailed overview of the intervention.
