# Supplementary material for: Assessing the scalability of an integrated falls prevention service for community-dwelling older people: a mixed methods study
Source: BMC Geriatr. 2022 Jan 3;22:17. doi: 10.1186/s12877-021-02717-6 (PMC8721469; doi:10.1186/s12877-021-02717-6)
Supplement: Supplementary file 1 — Additional file 1: S1. TIDieR-PHP intervention description. S2. Intervention Scalability Tool (ISAT) domains and objectives. S3. Interview questions. S4. Readiness assessment questions in online survey. [file 12877_2021_2717_MOESM1_ESM.docx]

**Supplementary File**

**S1: TIDieR-PHP intervention description**

| **Item** | **Item description** |
| --- | --- |
| **1. Brief name** | **Cork Integrated Falls Prevention Pathway** |
| **2. Why** | **Rationale**   - Falls are a common and often devastating problem for older people (65+), leading to significant morbidity, mortality and use of health services. - An estimated one in three people aged 65+ and one in two of those aged 80+ fall once a year. - A rising ageing population means that the impact of falls is likely to increase. - While a range of falls-related services exist in the region (southwest Ireland), considerable service shortcomings have been identified, including lack of primary prevention services targeting falls and a lack of clear clinical guidelines and referral pathways in primary care for falls. - As most falls result from multiple, interacting factors (including physical and environmental factors), a range of approaches is needed to prevent falls among community-dwelling older people.   **Aims**  The aims of the Cork Integrated Falls Prevention Pathway are to:   - Reduce the risk of falls and falls-related harms among community-dwelling older people in the region - Improve service provision by providing a continuum of services with a single point of referral   **Intended outcomes**   - Reduced number of emergency department falls presentations - Improved mobility and independence of older population - Improved and more coordinated service provision, including a greater emphasis on primary care and easier access for service users in appropriate settings |
| **3. What and how** | **What**  The pathway consists of a continuum of new and existing services to prevent, manage and treat falls:  *New*   - Single point of referral to the service managed by a coordinator - Multifactorial falls risk assessment clinics delivered in primary care/healthcare centres - Preliminary screening for falls incidence and risk in primary care (GPs and public health nurses)   *Existing*   - Specialist assessment and treatment clinics in hospital setting - Consultant-led clinics on falls-related issues (i.e. syncope and frailty) - Community exercise classes to promote improved strength and balance - Rehabilitation services delivered in clients’ homes and provided by a multidisciplinary team (CREST)   **How**   - Funding – the national health service (Health Service Executive or ‘HSE’) provided the funding to establish a falls pathway in the region with a dedicated Falls coordinator, clerical assistant and rehab assistant. - Steering committee – a steering committee was formed to oversee management and implementation of the pathway; this committee comprises health professionals and management working in older persons’ services and/or with an interest in falls prevention. - Service providers – the majority of service providers for this pathway are working in existing services (i.e. not newly recruited) and have been delegated to incorporate the falls component into their current work (e.g. multidisciplinary team providing a weekly falls risk assessment clinic) or were already providing services in this area (e.g. geriatric consultant). Only three new posts have been created (see previous point). - Service users – the target population for this service are recruited through referrals from other health professionals already working in the health system (e.g. GPs, public health nurses, emergency department professionals); the service is aimed at community-dwelling older people (as opposed to those in residential care) and mainly those aged 65+, although exercise classes are also accessible to younger age groups (50s+) to promote falls prevention. - Delivery – the service is delivered in a range of setting spanning primary and secondary care, community settings and home-based settings. - Legislation – the service is not underpinned by any legislation and is not a national service; however, it does align with national health service policy goals for older persons, e.g. in relation to providing an integrated model of care and prioritising falls prevention/management services as a key issue for older persons services. |
| **4. What materials** | **Informational materials:** Training for healthcare professionals (occupational therapists, physiotherapists, nurses) providing falls risk assessment clinics  **Administrative:** Falls coordinator to oversee service and manage/triage referrals; Administrative support to falls coordinator and falls risk assessment clinics  **Infrastructure:** Room space for FRAC, specialist clinics, coordinator/clerical officer and community exercise classes; Parking facilities for service users and staff  **Equipment:** Administration: computer, printer, photocopier, filing cabinet  For FRAC: Quick Screen assessment kits (e.g. visual chart, aerobic test, monofilament test, assessment forms), standard chair with arms for sit-stand test  For specialist clinics: access to cardiology (e.g. ECG), tilt table, monitors |
| **5. Who provided** | The services in the pathway are delivered by a range of professionals, the majority of whom are working in existing services, along with a number of newly created positions to coordinate the service:   - Falls service coordinator (with nursing or clinical background) - Administrator (to provide administrative support) - Multidisciplinary team (OTs, physiotherapists, nurses) to deliver the multifactorial risk assessment clinics - Geriatric consultant (x2) for specialist clinics - Exercise professionals or physiotherapists for exercise classes - GPs and public health nurses for preliminary screening - Rehab team for home rehabilitation service (CREST) (multi-disciplinary team comprising physiotherapists, occupational therapists, public health nurse, general nurse, rehabilitation assistants) |
| **6. Where** | The services are delivered in a range of settings in the region:   - Primary care settings – multifactorial falls risk assessment clinics are provided in primary care/healthcare centres in six locations in the region; initial screening for falls is also conducted in primary care settings, i.e. GP practices - Secondary care (hospital) settings – specialist falls risk assessment clinics (i.e. with consultant input) and geriatric clinics (one for syncope and one for frailty) are provided in a hospital setting - Community settings – exercise classes for strength and balance training are held in community centres or sports clubs - Home-based setting – rehabilitation services are provided by a multidisciplinary team in the service user’s own home |
| **7. When and how often** | The time and frequency of delivery varies depending on the service as follows:   - Falls risk assessment clinics are generally provided on a weekly basis (note: due to Covid-19, these have been temporarily suspended but are due to resume in the coming months) - Specialist falls assessment clinics (i.e. with consultant input) are also provided on a weekly basis - Specialist geriatric clinics (syncope and frailty clinics) are provided on a weekly basis (one on Tuesdays, one on Thursdays) by the two consultants involved - Exercise classes are generally run in six-week blocks on a weekly basis - GP/nurse screening for falls is recommended for all older persons (65+) once a year - Home-based services are provided on a needs basis |
| **8.1 Planned variation** | Variation planned for newly established falls risk assessment clinics is as follows:   - Timing – while the clinics are usually delivered on a weekly basis, the day of the week differs by clinic location and can be set according to health professionals’ availability and work schedule - Location – the clinics can be run in either a primary care centre, community centre or health centre; as a lot of the equipment used is portable, it does not necessarily need to be conducted in a primary care centre but could be held in a more generic location - Multifactorial assessment – the Quick Screen multifactorial assessment was the chosen standardised assessment; however, there is leeway to use another type of standardised assessment should the pathway be rolled out to other regions |
| **8.2 Unplanned variation** | This is not included in the intervention description as it pertains to findings of the evaluation research on implementation of the pathway |
| **9.1 How well** | The following strategies have been used to help maintain fidelity and support implementation:   - Appointing a dedicated Falls coordinator to act as a single point of contact to triage referrals into the pathway, ensuring appropriate referrals and more streamlined coordination - Establishing a steering group to guide and oversee implementation - Providing training, mentoring and coaching to health professionals running the falls risk assessment clinics to help ensure that they deliver the clinics as intended and to facilitate multidisciplinary working - Providing administrative support to facilitate the referral process and scheduling of appointments (dedicated full-time clerical officer) - Maintaining a database of referrals to the pathway, including data on onward referral and/or treatment. This enables the Falls coordinator to track the number of referrals into the pathway, whether the patient has received appropriate treatment/assessment, what type of treatment, etc. |
| **9.2 How well – delivery** | This is not included in the intervention description as it pertains to findings of the evaluation research on implementation of the pathway |

**S2: Intervention Scalability Tool (ISAT) domains and objectives**

| **Domain** | **Objectives** |
| --- | --- |
| **Part A** | |
| **A1: The problem** | Consideration of the problem that is being addressed. The questions in this domain seek a description of the problem, who it affects, what it affects and how it is currently being addressed (if at all). |
| **A2: The intervention** | Description of the proposed service to address the problem. |
| **A3: Strategic/political context** | Consideration of the current strategic/political/environmental contextual factors that are potentially important influences on the service to be scaled up. |
| **A4: Evidence of effectiveness** | Consideration of the level of evidence available to support the scale-up of the proposed service, such as scientific literature and/or other known evaluations of the intervention. |
| **A3: Intervention costs and benefits** | Consideration of the known costs of the service delivery as well as any quantifiable benefits. This includes the results of any types of economic evaluation studies. |
| **Part B** | |
| **B1: Fidelity and adaptation** | Consideration of whether there are any proposed changes to the service required for scale-up. |
| **B2: Reach and acceptability** | Consideration of the reach and acceptability of the intervention for the target population. |
| **B3: Delivery setting and workforce** | Consideration of the setting within which the intervention is delivered as well as the delivery workforce. |
| **B4: Implementation infrastructure** | Consideration of the potential implementation infrastructure required for scale-up. |
| **B5: Sustainability** | Consideration of the potential longer-term outcomes of the scale-up and how, once scaled up, the intervention could become sustainable over the medium to longer term. |

Source: Milat et al, 2020 [15]

**S3: Interview questions**

1. Firstly, what is **your role** in relation to this service?
2. Next, can you **describe the** service and what its purpose is?
3. Can you briefly describe the current **delivery settings for** the service and will the intervention be delivered in the same type of setting at scale?
4. How do you think the **Covid** pandemic will influence the service?
5. What **existed before** this service and what is the situation nationally regarding falls prevention services for older people?
6. What **level and type of scale-up** do you envisage for this service?
7. Do you think that any aspects of the service need to be **changed** before scale-up to other regions?

- If yes, what do you think needs to be changed and why?

- Would these changes affect the intended outcomes in any way?

- Are there parts or aspects of the pathway that you would consider essential and recommend to other areas and parts that are non-essential/would not recommend to other areas?

1. What is the **cost** of implementing the service – any indicators (e.g. staff costs, cost of training, infrastructure, equipment)?
2. Have there been any **unintended/adverse consequences** of the service so far?
3. Who are the **target population** for this service?

- How was this target population identified/recruited in the pilot?

- Any indicators so far of level of participation of target population during the pilot phase?

1. Will it be the same **target population** at scale?

- How will this target population be recruited at scale?

- How will you track this at scale-up?

- What do you think might be the barriers/facilitators to reaching the target population at scale?

1. Can you briefly describe the delivery **workforce** and **referrers** for this service and will the same workforce/referrers be used at scale-up?

- Do you think the pathway was acceptable to the delivery workforce and is likely to be acceptable if implemented at scale?

- What types of supports or resources were put in place to support the workforce in implementation?

1. In your experience, what type of **infrastructure** (e.g. clinic facilities, IT equipment) is needed to implement this service?
2. Do you have any sense of the **infrastructure** required **for scale-up** (including resources and timeframe needed to build this infrastructure)?

- Do you foresee any barriers/facilitators to building/acquiring this infrastructure at scale-up?

- What types of support do you think would be necessary for other sites intending to implement the service in the event of scale up?

1. Do you have any idea of the **projected timeframe** for scale-up of this service?

- Timeframe needed to achieve the required uptake/adoption by delivery organisations

- Timeframe needed to achieve the desired levels of resources/recruitment of delivery workforce

1. How are you currently monitoring **fidelity to the intervention** and how will you monitor this if scaled up? (fidelity being the extent to which parts of the falls prevention pathway and/or assessments are delivered as originally planned)
2. Once scaled up, how **sustainable** do you think the service would be in the medium to long term (5-10 years)?

- Will it require a large commitment of funds (initial or ongoing)? Have any other funding models for sustainability been considered?

- Is the proposed delivery workforce sustainable (in terms of supply or financially)? Have any other models for maintaining workforce been considered?

- What level of integration into existing service delivery settings or organisations will this pathway require if scaled up and is this sustainable?

1. Thinking about the implementation of this service, what key factors or **pre-conditions** do you think would need to be in place to enable the successful scale-up of this pathway to other sites or regions?
2. Do you think there is a strong **rationale** for scaling up this service?

**S4: Readiness assessment questions in online survey***

**Based on the readiness assessment questions provided in the ISAT* [16], *with the exception of Qs. 16 and 21 (Covid-related questions)*

**1. In your opinion, are falls among older people of sufficient priority to warrant scale up of the service to address this problem?** *(Please select one option)*

- Not at all
- To a very small extent
- Somewhat
- To a large extent
- Not applicable (i.e. not answerable based on the information available)

**Why have you selected the above response?** *(Please type your answer below – your feedback is greatly valued)*

| **2. Do you think the outcomes intended by this service address the needs of the target group (65+ years) and/or problem (falls and falls-related harms)?** *(Please select one option)* |
| --- |

- Not at all
- To a very small extent
- Somewhat
- To a large extent
- Not applicable (i.e. not answerable based on the information available)

**Why have you selected the above response?** *(Please type your answer below – your feedback is greatly valued)*

| **3. To what extent is addressing the problem of falls consistent with policy/strategic directions or priorities?** |
| --- |

*(Please select one option)*

- Not at all
- To a very small extent
- Somewhat
- To a large extent
- Not applicable (i.e. not answerable based on the information available)

| **4. Do you think scaling up the service would be strategically useful to funders/funding agency (i.e. HSE)?** |
| --- |

*(Please select one option)*

- Not at all
- To a very small extent
- Somewhat
- To a large extent
- Not applicable (i.e. not answerable based on the information available)

**Why have you selected the above responses?** *(Please type your answer below – your feedback is greatly valued)*

| **5. Based on the evidence available (above or elsewhere), do you think the falls service will be**  **effective in addressing the problem in the target population?** *(Please select one option)* |
| --- |

- Not at all
- To a very small extent
- Somewhat
- To a large extent
- Not applicable (i.e. not answerable based on the information available)

**Why have you selected the above response?** *(Please type your answer below – your feedback is greatly valued)*

| **6. Based on the evidence available, do you think that the benefits of the service could outweigh the costs?** |
| --- |

*(Please select one option)*

- Not at all
- To a very small extent
- Somewhat
- To a large extent
- Not applicable (i.e. not answerable based on the information available)

**Why have you selected the above response?** *(Please type your answer below – your feedback is greatly valued)*

**7. In your opinion, will the core components of the scaled up falls service be consistent with what was previously shown to be effective in the literature?** *(Please select one option)*

- Not at all
- To a very small extent
- Somewhat
- To a large extent
- Not applicable (i.e. not answerable based on the information available)

**8. If the core components of the service are to be changed/adapted from its original form during scale up, will the impact of the changes/adaptations likely be favourable?** *(Please select one option)*

- Not at all
- To a very small extent
- Somewhat
- To a large extent
- Not applicable (i.e. not answerable based on the information available)

**9. To what extent can service fidelity (i.e. whether the service is being delivered as intended) be monitored and/or maintained if implemented at scale?** *(Please select one option)*

- Not at all
- To a very small extent
- Somewhat
- To a large extent
- Not applicable (i.e. not answerable based on the information available)

**Why have you selected the above responses?** *(Please type your answer below – your feedback is greatly valued)*

**10. Do you think the service in its current form has the potential to reach the intended target population at scale?** *(Please select one option)*

- Not at all
- To a very small extent
- Somewhat
- To a large extent
- Not applicable (i.e. not answerable based on the information available)

**11. Do you think the service likely to be acceptable to the target population at scale up?**

*(Please select one option)*

- Not at all
- To a very small extent
- Somewhat
- To a large extent
- Not applicable (i.e. not answerable based on the information available)

**Why have you selected the above responses?** *(Please type your answer below – your feedback is greatly valued)*

**12. Is the delivery setting(s) selected to deliver the service at scale consistent with that used in previous studies and jurisdictions?** *(Please select one option)*

- Not at all
- To a very small extent
- Somewhat
- To a large extent
- Not applicable (i.e. not answerable based on the information available)

**13. Is the workforce intended to deliver the service at scale consistent with that used in previous studies?** *(Please select one option)*

- Not at all
- To a very small extent
- Somewhat
- To a large extent
- Not applicable (i.e. not answerable based on the information available)

**14. Is the service likely to be acceptable to the delivery workforce involved in its delivery at scale?**

*(Please select one option)*

- Not at all
- To a very small extent
- Somewhat
- To a large extent
- Not applicable (i.e. not answerable based on the information available)

**15. As the service requires integration into existing organisational and community structures, do you think this is likely to be feasible?** *(Please select one option)*

- Not at all
- To a very small extent
- Somewhat
- To a large extent
- Not applicable (i.e. not answerable based on the information available)

**16. In light of Covid-19, do you think it is feasible to scale up this service?** *(Please select one option)*

- Not at all
- To a very small extent
- Somewhat
- To a large extent
- Not applicable (i.e. not answerable based on the information available)

**Why have you selected the above responses?** *(Please type your answer below – your feedback is greatly valued)*

**17. Do you think the implementation infrastructure requirements for scale up will be feasible to acquire?** *(Please select one option)*

- Not at all
- To a very small extent
- Somewhat
- To a large extent
- Not applicable (i.e. not answerable based on the information available)

**Why have you selected the above response?** *(Please type your answer below – your feedback is greatly valued)*

**18. In your opinion, is the level of integration of the service into delivery settings required for implementation at scale sustainable?** *(Please select one option)*

- Not at all
- To a very small extent
- Somewhat
- To a large extent
- Not applicable (i.e. not answerable based on the information available)

**19. In your opinion, is the level of resourcing required to implement the service at scale sustainable?** *(Please select one option)*

- Not at all
- To a very small extent
- Somewhat
- To a large extent
- Not applicable (i.e. not answerable based on the information available)

**20. In your opinion, is the delivery workforce required for implementation at scale sustainable (both service delivery and coordination workforce)?** *(Please select one option)*

- Not at all
- To a very small extent
- Somewhat
- To a large extent
- Not applicable (i.e. not answerable based on the information available)

**21. In light of Covid-19, do you think implementation at scale of this service would be sustainable in the long term?** *(Please select one option)*

- Not at all
- To a very small extent
- Somewhat
- To a large extent
- Not applicable (i.e. not answerable based on the information available)

**Why have you selected the above responses?** *(Please type your answer below – your feedback is greatly valued)*
